# National landscape assessment of academic medical center support for expanded access to investigational products

**DOI:** 10.1017/cts.2022.494

**Published:** 2022-11-16

**Authors:** Misty Gravelin, Joan E. Adamo, Sharon Ellison, Erika Segear, Amanda B. Parrish, Christine Deeter, Jennifer Hamill, Laurie Rigan, George A. Mashour, Kevin J. Weatherwax

**Affiliations:** 1 Michigan Institute for Clinical and Health Research (MICHR), Michigan Medicine, University of Michigan, Ann Arbor, MI, USA; 2 Clinical & Translational Science Institute, University of Rochester Medical Center, Rochester, NY, USA; 3 Duke University School of Medicine, Durham, NC, USA; 4 Department of Anesthesiology, Michigan Medicine, University of Michigan, Ann Arbor, MI, USA; 5 College of Pharmacy, University of Michigan, Ann Arbor, MI, USA

**Keywords:** Expanded access, compassionate use, investigational drugs, investigational medical devices, CTSA, academic medical centers

## Abstract

Expanded access (EA) provides a pathway for the clinical use of investigational products (drugs, biologics, and medical devices) for patients who are without satisfactory therapeutic options and for whom a clinical trial is not available. Academic medical centers (AMCs) are likely to encounter EA requests, but it is unknown what support is available at these institutions for physicians seeking EA for patients. Methods: A landscape assessment was conducted at AMCs, focused on those within the Clinical and Translational Science Awards (CTSA) consortium. Results: Forty-seven responses were evaluated including 42 CTSA hubs. The large majority (43 of 47 respondents) reported using single-patient EA, while 37 reported multi-patient industry sponsored EA and 37 reported multi-patient investigator-initiated EA. Only half reported central tracking of EA requests. Support was available at 89% of sites for single-patient EA but less often for multi-patient EA. Types of support varied and were focused largely on the initial submission to the FDA. Conclusion: Use of and support for EA is widespread at AMCs, with support focused on single-patient requests. Gaps in support are common for activities after initial submission, such as FDA reporting and data collection.

## Introduction

The use of investigational products (drugs, biologics, and medical devices) is typically associated with clinical trials, where the purpose is to collect data evaluating the safety and efficacy of the test agent. There are, however, patients who may benefit from the use of an investigational product for a serious condition, but for whom there are no effective approved treatments or available clinical trials. For these patients, a physician can request the use of an investigational product through the FDA’s expanded access (EA) pathway. These requests can be for individual patient access including emergency use, compassionate use of devices, or single-patient investigational new drug (IND) applications. Intermediate-size patient population IND applications can be used to request access for small groups and physician/investigator-sponsored use as well as treatment protocols or large, industry-sponsored programs [1].

Due to their combined mission of research and clinical care, academic medical centers (AMCs) are likely to encounter EA requests. However, while these institutions have extensive infrastructure in place for clinical trials of investigational products, the same level of support for treatment with an investigational medical product under EA may not exist. The ability to support EA requests is an important part of accommodating patient needs at tertiary AMCs, where disease complexity and severity is high.

The Clinical and Translational Science Awards (CTSA) program was established by the National Institutes of Health (NIH) to help lower the barriers to transforming foundational discoveries into improved health. One focus of this consortium has been the development of regulatory support cores for clinical trials involving investigational products [2]. Since CTSA regulatory support cores have extensive experience in the FDA regulatory process for clinical trials, it stands to reason they might be called upon to facilitate EA requests. It has been shown that the level and types of support for clinical trials from these regulatory support cores vary across institutions, but nothing is known about the support for EA [3].

Transforming Expanded Access to Maximize Support and Study (TEAMSS) is an NIH-funded project to develop and disseminate best practices for EA through the CTSA consortium [4]. As a part of this effort, we conducted a landscape assessment of existing support for the EA process.

## Methods

The purpose of the landscape assessment was to determine the utilization of and support for the EA process in both CTSA-affiliated and other AMCs during a representative year.

A survey was developed with 35 questions, although not all questions were posed to all respondents due to branching logic. Questions were divided into five topics. The first section had questions related to the role of the respondent, the institution they were responding on behalf of, and whether they were associated with a CTSA hub. Section 2 dealt with the use of EA at the site, in terms of what type (drug or device; single patient, intermediate-size, or other multipatient treatment program) and how many requests were received. The number of requests could be expressed as an exact number or an approximate range. Questions on the IRB process were the focus of the third section and consisted of yes or no questions to gather information on whether the local IRB had specific EA workflows or resources. Section 4 broadened these questions to the level of information the institution had available, which included multiselect questions to identify which support services were available. The final section consisted of free text, long-answer questions addressing the development of support, benchmarking of support, and the challenges that EA presented to the physicians and to the institution.

The survey was placed on the University of Michigan Qualtrics platform. Efforts were made to reach all CTSA hubs, through an invitation sent to the PI of each CTSA hub by TEAMSS PI (GAM) and then to the administrative director by TEAMSS Co-PI (KJW) for each site. To reach the regulatory support staff most likely to have the information, a further invitation was extended to all members of a national IND/IDE workgroup focused around CTSA Regulatory Support Core staff. In addition, the survey was added to the Center for Leading Innovation and Collaboration (CLIC) website under the TEAMSS section with an open invitation to participate [5].

Reminder invitations were issued to all groups as well as personalized reminders to the PI at CTSA hubs that had not responded. The survey was open from 11/19/2019 to 3/5/2020 (last response).

Analysis was limited to descriptive characterization, with coding for themes found in the qualitative answers.

## Results

There were 57 unique responses, though not all respondents identified their institution. Several responses were excluded due to either having only one or two answers or being identified as a duplicate to another response (n = 10). If there were several responses from within a single hub or responses from multiple institutions within a hub, responses were combined into a single hub response if concordant. For hubs with affiliated institutions that had separate regulatory support, the responses were not combined. The results are reported as representing 42 CTSA hubs, three hub-affiliated institutions, and two non-CTSA sites, for a total of 47 evaluable responses. One of the non-CTSA sites was from an institution previously affiliated with a CTSA hub. It should be noted that not all respondents answered all parts of all questions. Some responses contained blank entries for specific questions, which were excluded in the evaluation of individual questions. For these questions, the total number of responses are indicated separately.

### Respondents

Within the current CTSA hub responses (n = 42), 29 were self-identified as coming from a regulatory support role within the hub (69%). Other respondents from the CTSA hubs identified as the following: principal investigator (three), IRB specialists (three), other roles within the institution (six), and no role identified (one).

### Use of Expanded Access

**Fig. 1.**
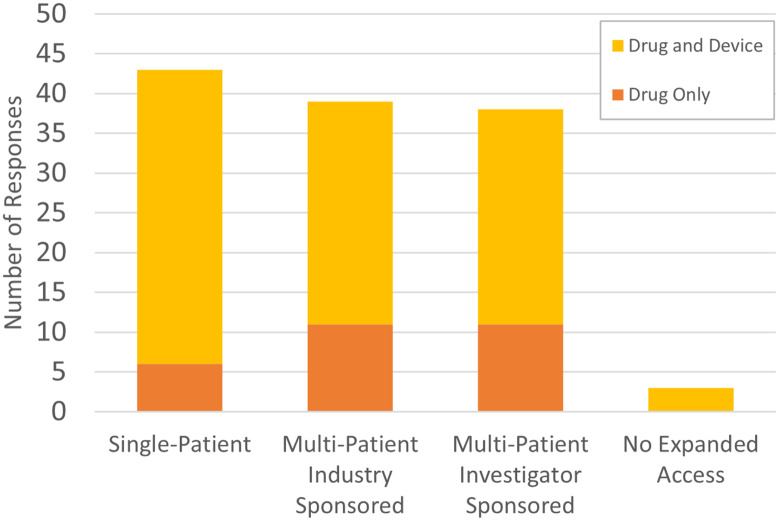
Use of expanded access.

When asked if physicians treat patients clinically through EA, most institutions indicated that they did. The responses were tallied under the categories of EA single patient (37 drugs and devices plus 6 drugs only; n = 44), multiple patient industry-sponsored (28 drugs and devices plus 11 drugs only; n = 44), and multiple patient physician/investigator-sponsored (27 drugs and devices plus 11 drugs only; n = 44). See Fig. 1. Three respondents reported that no EA was used at their institution (7%). One institution indicated that they used multi-patient industry-sponsored programs but not single-patient or investigator-initiated.

### Tracking

**Fig. 2.**
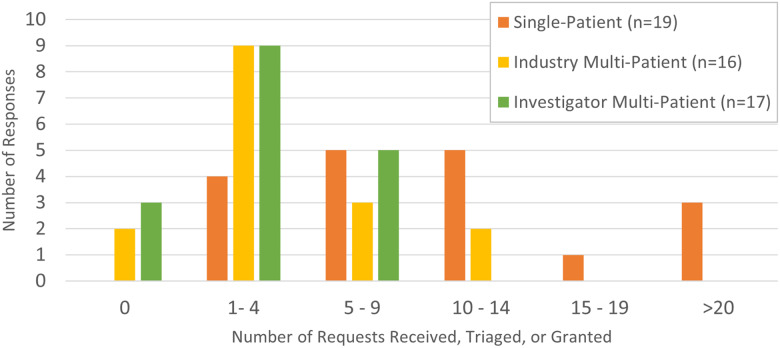
2018 expanded access requests reported.

Institutional tracking for EA submissions was not centralized or uniform. Only 23/45 (51%) indicated central tracking, with seven being centrally tracked by the CTSA hub. Twenty-one institutions indicated they had data for the calendar year 2018. These data are detailed in Fig. 2. It is notable that, among the 13 respondents that could provide the specific number of requests, there were a total of 199 single patient uses reported. For fiscal year 2018, the FDA received 1912 total single patient submissions to all centers [6].

Among the 23 respondents with centralized tracking at their institution, 17 (74%) indicated that this number had stayed the same or increased since the previous year, and none indicated that the number had decreased, which is consistent with the trend noted nationwide [6]. Of the nine who indicated an increase, the majority reported the magnitude of increase to be 25–49%.

### Institutional Review Board Process (IRB) for EA

Since the IRB is an integral part of institutional EA process, the survey queried the processes and resources provided for EA use. Twenty-two (49%; n = 45) respondents had specific IRB application types for at least one form of EA, which commonly included at least selections for emergency use (19/45) or single patient (15/45). One site reported a single patient application type only, and one indicated a multiple patient application type only. Six sites had distinct application types for all three EA types.

Twenty-three respondents reported that they had EA informed consent templates (51%; 23/45). Those with a specific application type and with an informed consent template did not fully overlap. Five sites reported no consent template despite a separate EA application type, and six had a consent template but no distinct application type.

Of the 23 responses without a separate submission application type for EA, 11 did and 11 did not have a means to specifically designate a submission as EA. One respondent reported that they did not know. Thirty-four sites reported specific IRB routing or procedure for EA; nine had no such procedure, and a further two did not know.

### Support for EA Submissions

Support for preparing and filing EA submissions was varied. When asked if physicians at the institutions submitted the EA requests on their own behalf, the majority, 78% (35/45), indicated that they did while 18% (8/45) reported that they did not submit their own requests. Two respondents did not know. Respondents were separately asked whether their institutions offered support for physicians doing EA submissions. Eighty-nine percent (39/44) reported that support was available, while 9% (4/44) had no support, and one respondent did not know. Of the 39 affirmative responses, seven indicated only direct CTSA hub support for the submissions, and 11 reported only support outside the CTSA; 16 selected that the institution had both CTSA and non-CTSA institutional support. Five reported other support that they felt did not qualify as CTSA or non-CTSA related for various reasons (for example, only consultation available, or support only available to select units). One respondent indicated that support was available but did not know which units provided it.

This support mirrored the reported submissions by category and type. Thirty-six offered support for single patient EA (100% of respondents to the question), of which three were only available for drugs and not devices. Industry-sponsored multipatient programs were supported by 27 sites (75%; four of which were drug only), and 32 (89%) had support for multiple-patient institution or investigator sponsored programs.

The specific types of support provided were relatively heterogeneous, as reported in Table 1. Common types of support were for initial FDA and IRB application submissions, followed by assistance with the informed consent, drug dispensing, and contracts. Fewer provided support for the postapproval-reporting requirements, particularly for the IRB, where less than half offered this kind of assistance. Only 27% offered support for data collection requirements, which are common among industry-sponsored multipatient EA, even though more than half of sites (27/36; 75%) offered some level of support for other aspects of EA.

**Table 1.** Institutional support for expanded access (n = 41)

Type of support	Support available	% of responses
Assistance with the Food and Drug Administration (FDA) submission	35	85
Assistance with the Institutional Review Board (IRB) submission	32	78
Development and preparation of the informed consent	29	71
Data collection requested by Expanded Access Program sponsors	11	27
Assistance with post-approval reporting requirements for FDA	24	59
Assistance with post-approval reporting requirements for IRB applications	16	39
Assistance with contracts with manufacturers	28	68
Assistance contacting manufacturers to make Expanded Access requests	22	54
Assistance creating a drug/device order and/or dispensing the investigational product	21	51
Research Pharmacy assistance for dispensing of investigational drugs	29	71
Assistance in clinical billing for the investigational product	19	46
Other support	8	20

Another question was asked about written guidance documents available from each institution. These responses are listed in Table 2. Guidance documents on emergency use was the only selection endorsed by a majority of respondents. Although assistance for contracts was available at the majority of sites, written guidance documents on this topic was only indicated by six respondents (Table 2).

**Table 2.** Written guidance (n = 39)

Type of guidance	Guidance available	% of responses
Emergency use	33	85
Food and drug administration submission	15	38
Institutional review board submission	22	56
Contracts	6	15
Informed consent	21	54
Physician responsibilities	17	44
Other or not otherwise specified	7	18

### Obstacles to EA Use

A free-text question asked respondents to identify the key obstacles to the use of EA at their institution. The most frequently cited concern was knowledge of the process by the treating physicians, which was mentioned in 15 comments. This included a lack of understanding of what EA is, when a patient qualifies, what institutional or regulatory procedures are required, what support was available, or misunderstanding if a product was available through EA. Issues with the institutional process, or lack thereof, were referenced in 13 responses, often focusing on a lack of coordination between units (departments, divisions, offices, or other administrative subsets) or a lack of centralized resources, as well as restrictions on support to certain units such as those within a Comprehensive Cancer Center. Several also addressed specific institutional challenges in contracts, IRB process, or compliance.

While most responses discussed obstacles to single-patient use of EA, three specifically indicated the issues presented in coordination of multipatient programs, particularly industry-sponsored programs. Although a small portion of the overall respondents, these comments were similar in their concerns: the increased overlap with research, the need for similar infrastructure to a clinical trial, and the lack of appropriate funding to cover costs.

## Discussion

This national survey revealed that EA support systems are widespread among AMCs, although such support is highly heterogenous. Assistance was widely available for single patient cases, which may reflect the efforts of the FDA since 2016 to raise awareness of this pathway and to make it more accessible [7,8]. Resources and support were more focused on the activities required to obtain investigational products and initiate treatment (i.e., preparing submissions, contracts, and informed consents) but less available for the regulatory requirements to maintain applications, such as annual reports and withdrawals. Assistance was offered by the fewest institutions for items associated with the ongoing support of multi-patient EA, such as data collection. Despite the infrastructure this implies, respondents voiced concern that physicians were not aware that support was available. Further, the number of blank and “I don't know” answers may imply that even among those staff surveyed, the totality of resources available for EA may not be well-known.

The data from this survey show the overlap between research and treatment use. While the distinction is clear in principle, the processes to advance EA still require research infrastructure, which promotes conflation between treatment and research. Clinicians who do not normally conduct clinical research are stymied by the research-related requirements for treatment use, an obstacle specifically called out in many comments. Neither single- nor multi-patient EA is funded to the level of an equivalent clinical trial, which creates additional barriers for entry into the research ecosystem.

Although multi-patient programs have a long history [9], they exist in somewhat of a vacuum related to process guidance or literature. The available FDA guidance, “Treatment Use of Investigational Drugs: Guidance for Institutional Review Boards and Clinical Investigators,” was published in 1998 and only discusses the minimal requirements of these INDs [10]. This is compared to single patient EA, for which the FDA has provided guidance for expedited IRB review for both emergency and nonemergency requests [7,9]. As a result, these programs can vary from minimally intensive umbrella INDs used only to provide drug for treatment under a physician’s discretion, to prescriptive protocols with data collection that may be indistinguishable from an open label clinical trial. Many survey respondents mentioned that these programs, much more than single patient cases, were handled in the same manner as other clinical trials, which may be due to this “research-like” quality.

However, the challenges mentioned by respondents often speak to the imperfect fit for these multi-patient programs in the research enterprise. These include issues of funding or a mismatch between the purpose of treatment and the reality of participation in a program. This is also seen in the aggregated responses. Although more than half of institutions provided some degree of support for multi-patient programs (68% for investigator initiated; 57% for industry sponsored), specific resources were rare. Only 36% of respondents reported an IRB process for multi-patient programs, and fewer (16%) of sites reported a specific IRB application type. This implies that multi-patient programs at the remaining sites must be handled through another mechanism. Institutional support for activities that may be required by sponsors is similarly low, with 27% (11 sites) reporting support for data collection.

The use of research infrastructure may be a reasonable approach if multi-patient EA programs were rare, but the majority of respondents reported using these programs to provide treatment at their site (83% for industry sponsored; 81% for investigator initiated). Further, both the FDA and drug manufacturers want to increase the use of these programs, specifically because they allow for considerations such as data collection. To this end, the “Accelerating Access to Critical Therapies for ALS Act,” signed into law on December 23, 2021, specifically allocates funding for “purposes of scientific research utilizing data from EA to investigational drugs for individuals who are not otherwise eligible for clinical trials for the prevention, diagnosis, mitigation, treatment, or cure of amyotrophic lateral sclerosis” and to support research on other rare neurodegenerative diseases [11]. Thus, demand for support in these areas will likely grow.

### Limitations

Limitations of this landscape assessment relate to the population surveyed, the time frame, and survey design.

The survey was primarily addressed to AMCs with a CTSA hub, with 45/47 respondents affiliated with such an institution. Academic medical centers that are equipped to apply for and obtain these research infrastructure grants may be fundamentally different from those that are not, which would limit generalizability. Further, some findings may specifically reflect the focus of CTSA hubs on investigator-initiated research, including the higher level of support for physician/investigator-initiated multi-patient programs even though industry-sponsored programs were overall more prevalent.

Second, this survey addressed institutional support prior to the COVID-19 pandemic. The necessity of EA to provide treatment during this public health emergency may have led to changes in support. While these survey results may not be generalizable to the time of the pandemic, it appears unlikely that major structural changes at these institutions have occurred that would limit generalizability. As has been previously reported, use of EA may have increased at CTSA hubs, but resources for support did not [12].

As the intent of this survey was a broad consideration of use and availability, the study was not designed to provide a baseline assessment of how this support is provided, what funding provides for it, or the timelines for and outcomes of requests. The data we report on the frequency of use, in conjunction with the gaps in EA support and consequences of its use (including real costs and potential for noncompliance), represent an opportunity for future research.

Finally, there are possible limitations to interpretation based on the survey design. Not all questions required a response and, in the case of multi-select questions, it could be unclear whether the response was intended to be “none of the above” or blank. Some questions were beyond the knowledge of respondents, and “Do Not Know” responses were common. This accounts for the discrepancy between responses to a question and the total number of respondents.

## Conclusion

Expanded Access is meant to provide treatment to patients with serious or life-threatening conditions when other therapies are insufficient. At least within CTSA hubs, use of and support for some forms of EA is widespread. However, multi-patient programs appear to be under-resourced, which may present challenges as more therapies become available under these protocols.

## References

[1] Food and Drug Administration. *Expanded Access*, 2022. (https://www.fda.gov/news-events/public-health-focus/expanded-access)

[2] Zerhouni EA. Translational research: moving discovery to practice. Clinical Pharmacology & Therapeutics 2007; 81(x1): 126–128. 17186011 10.1038/sj.clpt.6100029

[3] Berro M , Burnett BK , Fromell GJ , et al. Support for investigator-initiated clinical research involving investigational drugs or devices: the Clinical and Translational Science Award experience. Academic Medicine 2011; 86(2): 217–223. 21169787 10.1097/ACM.0b013e3182045059 PMC13220793

[4] National Institutes of Health NRPORT. *Transforming Expanded Access to Maximize Support & Study*, 2020. (https://projectreporter.nih.gov/project_info_description.cfm?aid=9597774&icde=42762464&ddparam=&ddvalue=&ddsub=&cr=1&csb=default&cs=ASC&pball)

[5] Center for Learning Innovation & Collaboration. *TEAMSS*, 2022. (https://clic-ctsa.org/groups/teamss)

[6] U.S Food & Drug Administration. *Expanded Access (compassionate use) Submission Data*, 2021. (https://www.fda.gov/news-events/expanded-access/expanded-access-compassionate-use-submission-data)

[7] U.S. Food & Drug Administration. *Expanded Access to Investigational Drugs for Treatment Use – Questions and Answers. Guidance for Industry*, June 2016.

[8] Government Accountability Office. *Expanded Access Program Report*, May 2018.

[9] Expanded availability of investigational new drugs through a parallel track mechanism for people with AIDS and other HIV-related disease--PHS. Notice final policy statement. Federal Register 1992; 57(73): 13250–13259. 10118459

[10] U.S. Food & Drug Administration. *Treatment Use of Investigational Drugs. Guidance for Institutional Review Boards and Clinical Investigators*, January 1998.

[11] *Accelerating Access to Critical Therapies for ALS Act, HR 3537, 117th Cong*, 2021. (https://www.congress.gov/bill/117th-congress/house-bill/3537)

[12] Gravelin M , Wright J , Holbein M , et al. Role of CTSA institutes and academic medical centers in facilitating preapproval access to investigational agents and devices during the COVID-19 pandemic. Journal of Clinical and Translational Science 2021; 5(1): 1–28. DOI 10.1017/cts.2021.15. PMC8134898 34192051

